# Supplementary material for: Barriers to osteopathic manipulative medicine use: A qualitative study of physician attitudes and experiences
Source: PLoS One. 2025 Aug 11;20(8):e0330219. doi: 10.1371/journal.pone.0330219 (PMC12338785; doi:10.1371/journal.pone.0330219)
Supplement: S1 File — (DOCX) [file pone.0330219.s001.docx]

Protocol Title: A Mixed Methods Analysis of Barriers to Osteopathic Manipulative Medicine Services

IRB #: 23-004379

Principal Investigator: Stephen K Stacey, DO

You are being asked to participate in a research study about attitudes regarding osteopathic manipulative medicine (OMM) services. We plan to assess provider attitudes regarding osteopathic manipulative treatments (OMT), and to identify barriers to OMT referrals. We plan to interview all providers at Mayo Clinic and Mayo Clinic Health Systems who responded to our first survey and indicated they would be willing to be contacted for further questions or clarification. We want to determine:

1. The reasons why DOs do not choose to practice OMT.
2. Identify what kinds of patients MDs typically refer for OMT, if any.
3. Barriers to MD and DO referrals for OMT.

If you agree to participate you will be asked to complete a brief interview. The interview should last about 30 to 40 minutes and will be recorded and transcribed. None of your personally identifiable information will be maintained beyond the duration of this research study. You will receive no payment for your participation. Your current and future employment, education, or medical care at Mayo Clinic will not be affected by whether or not you participate. You may refuse to answer any question(s) you do not wish to answer. As with all research, there is a chance that confidentiality could be compromised; however, we take precautions to minimize this risk. Your information collected as a part of this research could be used for future research or distributed to another investigator for future research without additional informed consent from you, only after information that identifies you is removed.

Please understand your participation is voluntary and you have the right to withdraw your consent or discontinue participation at any time without penalty. Specifically, your current or future medical care at the Mayo Clinic will not be jeopardized if you choose not to participate.

If you have any questions about this research study you can contact me at [stacey.stephen@mayo.edu.](mailto:stacey.stephen@mayo.edu) If you have any concerns, complaints, or general questions about research or your rights as a participant, please contact the Mayo Institutional Review Board (IRB) to speak to someone independent of the research team at 507-266-4000 or toll-free at 866-273-4681.
