# Supplementary material for: Barriers to osteopathic manipulative medicine use: A qualitative study of physician attitudes and experiences
Source: PLoS One. 2025 Aug 11;20(8):e0330219. doi: 10.1371/journal.pone.0330219 (PMC12338785; doi:10.1371/journal.pone.0330219)
Supplement: S2 File — (PDF) [file pone.0330219.s002.pdf]

Study : *Exempt*

Summary

	Author:	Christina Dobrovolny (RS-Research Quality)
Jun16**2023**	Logged For (Application):	A Mixed Methods Analysis of Barriers to Osteopathic Mani[illegible]
	Activity Date:	6/16/2023 2:57 PM

Form

Send Notification for Application:

Notify All Study Staff:

Study ID:
23-004379

Full Study Title:
A Mixed Methods Analysis of Barriers to Osteopathic Manipulative Medicine

Application Type: Exempt

*** IRB Determination On The Level Of Risk For This Study:**

Minimal Risk

Greater Than Minimal Risk

Not Applicable

*** FDA Regulated:**

Yes

No

*** Motion:**

Approve

Not Human Subjects Research

Not Research

Exempt

Grant

*** Notification to PI:**

The above referenced application was reviewed by expedited review procedures and is determined to be exempt from the requirement for IRB approval (45 CFR 46.104d, Categories 2 and 4). Continued IRB review of this study is not required as it is currently written. However, any modifications to the study design or procedures must be submitted to the IRB to determine whether the study continues to be exempt.

The oral consent script and interview guide were reviewed and noted. As protected health information is not being requested from subjects for the interviews, HIPAA authorization is not required in accordance with 45 CFR 160.103.

The Reviewer approved waiver of HIPAA authorization in accordance with applicable HIPAA regulations for the chart review portion.

AS THE PRINCIPAL INVESTIGATOR OF THIS PROJECT, YOU ARE RESPONSIBLE FOR THE FOLLOWING RELATING TO THIS STUDY.
1) When applicable, use only IRB approved materials which are located under the documents tab of the IRBe workspace. Materials include consent forms, HIPAA, questionnaires, contact letters, advertisements, etc.
2) Submission to the IRB of any modifications to approved research along with any supporting documents for review and approval prior to initiation of the changes.
3) Submission to the IRB of all Unanticipated Problems Involving Risks to Subjects or Others (UPIRTSO) and major protocol violations/deviations within 5 working days of becoming aware of the occurrence.
4) Compliance with applicable regulations for the protection of human subjects and with Mayo Clinic Institutional Policies.

If Applicable, Set Expiration Date:

Additional Recipients of Notification:

There are no items to display

Attachments:

Name	Version	Date Created	Date Modified
There are no items to display			

*** Has a Certificate of Confidentiality Been Obtained for this protocol?**

Yes No

If there is a Certificate of Confidentiality, provide Expiration Date:

*** Consent Type:**

Exempt

Close
