# Supplementary material for: Barriers to osteopathic manipulative medicine use: A qualitative study of physician attitudes and experiences
Source: PLoS One. 2025 Aug 11;20(8):e0330219. doi: 10.1371/journal.pone.0330219 (PMC12338785; doi:10.1371/journal.pone.0330219)
Supplement: Attachment — Submitted filename: PLOS ONE Review Manuscript PONE-D-25-16174 OMT Barriers.docx [file pone.0330219.s003.docx]

PLOS ONE Review Manuscript PONE-D-25-16174: OMT Barriers.docx

May 20, 2025

Reviewer Notes

Stacey SK, Prins KM, Anderson SA, Quartiroli A. Barriers to Osteopathic Manipulative Medicine Use: A Qualitative Study of Physician Attitudes and Experiences

Abstract

The authors state that OMT is underutilized by DOs in spite of being an evidence-based treatment for chronic pain. The reader is then poised to look for reasons for this underutilization in a chronic pain patient population who apparently might benefit from OMT. Instead, the researchers conducted a survey of osteopathic physicians to find out why they do or do not use OMT, regardless of what patient population they serve.

Perhaps a bridging statement is needed to keep this line of thinking somewhat connected. Such as, “osteopathic physicians use OMT for a variety of common ailments, including acute neck and back pain, respiratory conditions, gastrointestinal dysfunctions and headaches.”

In the conclusion section of the abstract, the authors state that Time constraints, … and limited patient awareness emerged as significant barriers, along with patient panel management and practice autonomy. The last sentence then states that these barriers could be addressed through “streamlined scheduling” which addresses the scheduling barrier, and “enhanced OMT training, and advocacy for practice integration” which address which significant barrier? How do these last two suggestions address the patient awareness issue? Or the patient panel management issue? Or the practice autonomy issue? There is again a disconnect for the reader who is waiting to hear about future research and recommendations to address the barriers found.

Previous studies have noted that increasing patient awareness through public relations, increasing time allotments for patient visits for OMT, increase reimbursement for OMT, and expand the evidence base for OMT for other conditions besides chronic pain.

It would have been of interest to readers of the article to mention how many chronic pain patients are seen at the Mayo Clinic Health Systems, and how many actually receive OMT? How many are using opioids chronically?

Background

The first sentence can use a reference. Recommend using Starr, Healy and or Stacey:

Starr EG, Smith JF, Hanson RB, Woolstenhulme JB, Roush AJ, Sperry NB, Wilde B, Brooks AE, Zapata I. [Utilization and reimbursement trends of osteopathic manipulative treatment for Medicare patients: 2000-2019.](https://pubmed.ncbi.nlm.nih.gov/36996336/) J Osteopath Med. 2023 Mar 31;123(6):309-315. doi: 10.1515/jom-2022-0174. eCollection 2023 Jun 1.PMID: 36996336

Healy CJ, Brockway MD, Wilde BB [Osteopathic manipulative treatment (OMT) use among osteopathic physicians in the United States.](https://pubmed.ncbi.nlm.nih.gov/33512391/) J Osteopath Med. 2021 Jan 1;121(1):57-61. doi: 10.1515/jom-2020-0013.PMID: 33512391

Stacey S K, Harmelink B, Gordon B, et al. (May 08, 2025) A Retrospective Multicenter Analysis of Osteopathic Manipulation in Academic and Community Settings. Cureus 17(5): e83755. doi:10.7759/cureus.83755 (Mayo clinic)

The reference to Healy is used for the second to last sentence, so perhaps consider using the other two for the first sentence.

The second paragraph is weakly written. To strengthen the points the authors could consider as a second sentence something to the effect of: “one of the defining characteristics of osteopathic medical practice since its inception is the integration of OMT in a variety of clinical settings to alleviate somatic dysfunction, by itself, or in addition to patient education, medication and surgery in the care of the patient.”

The last sentence in the second paragraph also needs a reference. Stacey from the Mayo Clinic would be a good one here.

Results Section

In the PLOS ONE instructions to reviewers, they mention that the results of the data should be accessible to readers/researchers. How can the authors make the data and results available from interviews? There should be a way others can access the transcripts of the interviews, perhaps on a web site?

Discussion

The first paragraph alludes to “broader application of osteopathic principles”, but readers in general, those outside of the osteopathic education system or profession, would not likely comprehend the connotations of that reference to osteopathic principles. The authors should either expound on what they are and how this study applies to them, or drop it altogether as it has no bearing on the results or discussion section otherwise.

The authors use the word “gap” many times, and it might be easier on the reader if synonyms could be used so the reader is not inundated with a variety of “gaps” of different types and magnitudes. They mention a gap in understanding, a gap in utilization, a gap in education and training, a gap in patient access….

Conclusion

The data do not support the conclusion that patients aren’t aware. Patients were not interviewed in this study. In fact, one of the DOs interviewed stated that patients would accept OMT if it was explained to them what it does, in cases when the patient doesn’t know about it beforehand. So patient awareness may not be such a significant barrier. Certainly this study is not designed to assess patient awareness and thus it should not be part of the conclusion.

General

The remainder of the article is well conceived, written clearly, a pleasure to read, and the references are appropriate and up to date, other than the couple mentioned above. Methods were well articulated and referenced. The transcripts are the raw data and should be made available if another researcher wants to replicate or interpret the study in a different light, as the presentation of the data, the analyses and tables were anecdotal and thematic, with some examples selected by authors to make a point. Not sure if another researcher would come up with the same conclusions as these authors. Still, the article could benefit from some minor revisions as suggested above. It otherwise makes a scholarly addition to the literature on the subject.
