# Supplementary material for: Barriers to osteopathic manipulative medicine use: A qualitative study of physician attitudes and experiences
Source: PLoS One. 2025 Aug 11;20(8):e0330219. doi: 10.1371/journal.pone.0330219 (PMC12338785; doi:10.1371/journal.pone.0330219)
Supplement: Attachment — Submitted filename: Response to Reviewers.docx [file pone.0330219.s004.docx]

PLOS ONE Review Manuscript PONE-D-25-16174: OMT Barriers.docx

May 20, 2025

| **Reviewer comments** | **Author response** |
| --- | --- |
| **Abstract** |  |
| The authors state that OMT is underutilized by DOs in spite of being an evidence-based treatment for chronic pain. The reader is then poised to look for reasons for this underutilization in a chronic pain patient population who apparently might benefit from OMT. Instead, the researchers conducted a survey of osteopathic physicians to find out why they do or do not use OMT, regardless of what patient population they serve. Perhaps a bridging statement is needed to keep this line of thinking somewhat connected. Such as, “osteopathic physicians use OMT for a variety of common ailments, including acute neck and back pain, respiratory conditions, gastrointestinal dysfunctions and headaches.” | The reviewer brings up a good point that the introductory sentence sets up the reader for considering the wrong problem. We appreciate the suggestion the reviewer made, and have altered the first line of the abstract. |
| In the conclusion section of the abstract, the authors state that Time constraints, … and limited patient awareness emerged as significant barriers, along with patient panel management and practice autonomy. The last sentence then states that these barriers could be addressed through “streamlined scheduling” which addresses the scheduling barrier, and “enhanced OMT training, and advocacy for practice integration” which address which significant barrier? How do these last two suggestions address the patient awareness issue? Or the patient panel management issue? Or the practice autonomy issue? There is again a disconnect for the reader who is waiting to hear about future research and recommendations to address the barriers found. | The point is well received that this article focuses on how DOs conceptualize the barriers, and does not provide empiric support for any proposed solutions. At the same time, readers often look for some practical takeaway from the findings of the study. It appears that the particular solutions we suggested may drive attention away from the central topic of the research, so we rephrased the final sentence of the abstract. Rather than giving specific suggestions for how to improve access to OMM, it now simply advocates for intentional support from institutional leadership. |
| Previous studies have noted that increasing patient awareness through public relations, increasing time allotments for patient visits for OMT, increase reimbursement for OMT, and expand the evidence base for OMT for other conditions besides chronic pain. | These are good points. The nature of the present research is to map the thoughts, beliefs and opinions of osteopathic physicians and not to verify the accuracy of those opinions. Also, patients were not involved in the interviews of the present study. |
| It would have been of interest to readers of the article to mention how many chronic pain patients are seen at the Mayo Clinic Health Systems, and how many actually receive OMT? How many are using opioids chronically? | This could definitely be interesting data to help contextualize the practice patterns of the institution that was studied. Unfortunately we do not have access to the data suggested by the reviewer, and it was beyond the scope of this research to obtain that data. |
| **Background** |  |
| The first sentence can use a reference. Recommend using Starr, Healy and or Stacey:  Starr EG, Smith JF, Hanson RB, Woolstenhulme JB, Roush AJ, Sperry NB, Wilde B, Brooks AE, Zapata I. [Utilization and reimbursement trends of osteopathic manipulative treatment for Medicare patients: 2000-2019.](https://pubmed.ncbi.nlm.nih.gov/36996336/) J Osteopath Med. 2023 Mar 31;123(6):309-315. doi: 10.1515/jom-2022-0174. eCollection 2023 Jun 1.PMID: 36996336  Healy CJ, Brockway MD, Wilde BB [Osteopathic manipulative treatment (OMT) use among osteopathic physicians in the United States.](https://pubmed.ncbi.nlm.nih.gov/33512391/) J Osteopath Med. 2021 Jan 1;121(1):57-61. doi: 10.1515/jom-2020-0013.PMID: 33512391  Stacey S K, Harmelink B, Gordon B, et al. (May 08, 2025) A Retrospective Multicenter Analysis of Osteopathic Manipulation in Academic and Community Settings. Cureus 17(5): e83755. doi:10.7759/cureus.83755 (Mayo clinic)  The reference to Healy is used for the second to last sentence, so perhaps consider using the other two for the first sentence. | This is a good suggestion. The references have been updated for the first sentence to reflect the support for this statement from the Starr and Stacey articles. |
| In the background, it would have been good to temper the efficacy of osteopathy for chronic pain, depending on the type of pain and treatment. Some studies mention the absence of efficacy and the meta-analyses cited by the authors are not clear-cut on efficacy either or focus on specific patients. For example, Franke 2017 focuses on pregnant and post-partum women, which does not seem too relevant as a reference for chronic pain. Licciardone et al. is not a systematic review but a narrative review and cannot support efficacy. | It is beyond the scope of this current article give an in-depth review of the evidence surrounding OMM, though we wanted to set up the importance of exploring barriers to OMM. The second sentence contextualizes that OMM can be useful for several chronic pain conditions rather then chronic pain writ large, so this was brought into the first sentence. |
| The authors wrote: “However, the increasing prevalence of DOs in the US physician workforce has not led to a substantial increase in the use of OMM.” Since it is the reason why they ran their study, a reference must be added. | Citation added at the end of that sentence. |
| The authors wrote: “Barriers such as time constraints, lack of proficiency, insufficient reimbursement, and unsupportive work environments likely contribute to this underuse. » So they answered the question before doing the study? | The potential barriers we listed in the background are from the referenced studies. They help contextualize the findings from the present study. |
| The second paragraph is weakly written. To strengthen the points the authors could consider as a second sentence something to the effect of: “one of the defining characteristics of osteopathic medical practice since its inception is the integration of OMT in a variety of clinical settings to alleviate somatic dysfunction, by itself, or in addition to patient education, medication and surgery in the care of the patient.” | The reviewer pointed out an addition that helps contextualize the background and nature of OMM. This has been added to the second paragraph of the background. |
| The last sentence in the second paragraph also needs a reference. Stacey from the Mayo Clinic would be a good one here. | The citations for this sentence have been updated per the reviewer’s suggestion. |
| The objective is unclear. Several objectives are presented. Is to explore “the perspectives of DOs through indepth interviews and thematic analysis of their decision-making regarding the integration of OMM into their clinical practice” ou to provide “solutions to barriers they identified based on their training and professional experiences”? Both objectives are present in introduction. Since no solution are provided, the second sentence has to be modified. | We appreciate this perspective. The final paragraph of the background section has been modified to clarify the purposes of the study. |
| **Methods** |  |
| The paper should be written according to the COREQ checklists (see Equator). This is not the case here, and the information is poorly organized and hard to follow. In addition, a lot of information is missing that is mandatory according to COREQ and to be able to reproduce the study. The following information must be added. - What were the researcher’s credentials? E.g. PhD, MD - Participant knowledge of the interviewer - What was their occupation at the time of the study? - Was the researcher male or female? - What experience or training did the researcher have? - What did the participants know about the researcher? e.g. personal goals, reasons for doing the research - Was anyone else present besides the participants and researchers during the itw? - Were repeat interviews carried out? If yes, how many? - Were field notes made during and/or after the interview? - The interview guide must be included. - The coding tree must be included. - Were transcripts returned to participants for comment and/or correction? - What software, if applicable, was used to manage the data? - Did participants provide feedback on the findings? In results: - Data saturation must be discussed - The authors used the abductive thematic analysis so the Thematic Network Analysis figure must be included. - What was the duration of the interviews? - The theme covers (percents for each theme and each participant). | The information requested by the reviewer was inserted in numerous locations throughout the manuscript. These led to substantial revisions that we hope will be helpful to the reader. In particular, the COREQ guideline has now been used and explicitly referenced in the manuscript in the last sentence of the methods. The methods and results sections have numerous changes as outlined in the COREQ checklist and reprinted by the reviewer. |
| It is not clear if the pilot interviews have been added in the final analysis or not: - If yes, authors must remove the pilot interviews from the analysis: pilot data cannot be added, they are done to test, not to analyse. - If not, authors must detail the recruitment, the case data, etc. of these DO. Authors said: “3 investigators (S.K.S., K.M.P., and S.A.A.) analyzed each transcribed interview. » Each investigators analyzed all 16 itw or only some? | Two comments made here: Clarified that pilot interviews were not included in the final analysis, in the third paragraph of the methods. In the results we now clarify that “Each interview transcript was independently coded by the two investigators who had not conducted the interview.” |
| **Results** |  |
| In the PLOS ONE instructions to reviewers, they mention that the results of the data should be accessible to readers/researchers. How can the authors make the data and results available from interviews? There should be a way others can access the transcripts of the interviews, perhaps on a web site? | See reply under Editor Comments, below. |
| There are not enough interviewees’ quotes. The authors do not provide verbatim transcripts, so there is a need for many more quotes to know whether these are really the respondents' ideas or those of the authors. At least, one or two complete quotes must be added after each paragraph. For example, for System theme, the first two paragraphs do not quote any itw. What's more, the rare quotes are not accompanied by the interviewee's number. So we don't know the profile of the DO giving this or that information. | Participant number was added to the demographics table and quotes were identified by participant number. Numerous additional quotes were added throughout the results section to support the conclusions. |
| **Discussion** |  |
| The first paragraph alludes to “broader application of osteopathic principles”, but readers in general, those outside of the osteopathic education system or profession, would not likely comprehend the connotations of that reference to osteopathic principles. The authors should either expound on what they are and how this study applies to them, or drop it altogether as it has no bearing on the results or discussion section otherwise. | This is a good point, especially since the prior sentence doesn’t refer to principles per se, but to practice. Since the point of this manuscript is to explore barriers to the *practice* of OMM, the word “principles” was changed to “practice”. |
| 1. The authors use the word “gap” many times, and it might be easier on the reader if synonyms could be used so the reader is not inundated with a variety of “gaps” of different types and magnitudes. They mention a gap in understanding, a gap in utilization, a gap in education and training, a gap in patient access….  2. Also, there are several references about a gap, of different types and breadths, which is an overused term in this manuscript. | Where the term “gap” was used in the background, it was changed when doing so improved clarity. The reworking of the discussion led to the term “gap” being removed entirely. |
| In introduction, authors say that solutions need to be put in place, and I would have liked to have found some in the discussion. The discussion needs to be completed. Actually, it simply repeats the results without really delving into literature. For example, the fact that the interviewees were mainly MD from the Mayo clinic introduces a selection bias. I assume that the patients who come to the clinic are not the same patients as those who come to family practice where the practice of OMM will be different and easier. The responses give the feeling that the interviewed DO do not take care of patients who have come for osteopathic treatment but who have come for medical treatment. This is confirmed by the fact that DOs need their Leadership's approval to practice OMM. This in fact affects the other themes, such as Capability (if they don't practice much, they lose confidence in themselves). All this needs to be discussed. The results are only applicable to osteopaths practicing in clinics as MD clinicians. One of the respondents pointed out that in family medicine or primary care, OMM would be much more widely used. The fact that samples were ODs in a clinic setting is barely mentioned, although this should be specified in the title. Moreover, the authors compare the study to an emergency department study (the study states this in its title). Nor is there any discussion of the study's biases and limitations. | We substantially reworked the discussion to reflect the proposed solutions highlighted by our findings. We expanded on the limitations paragraph, noting “These results, while consistent across interviewees, represent only a select portion of DOs at a single institution where patients typically see DOs for conventional care rather than OMM per se.” and that “these findings may be most applicable to DOs practicing in large, multispecialty settings.”  Of note, DOs do not require leadership approval to practice OMM, so we clarified by adding the phrase “This type of hierarchy is typical of large organizations and may limit opportunities for OMM unless explicit institutional support is in place. This likely contributes to the perception that DOs must be “allowed” by the institution to use OMM.”  We also added a paragraph about suggestions for future research near the end of the discussion. |
| The authors talk about solutions in the introduction as one of the objectives, but no concrete solutions are provided and there are only a few very general sentences at the end of the discussion. The solutions need to be developed to make the work interesting. | This reviewer made similar helpful comments as the reviewer in the preceding comment, and these points are addressed there. |
| Table 2. “Training and regular practice” is displayed in Capability theme and not in Training theme. Why? Ces 2 themes véhiculent les mêmes idées: “level of comfort and proficiency » and « their feelings of capability when providing OMM” looks like similar. It's a bit the same for the System and Resources themes, where the ideas of organizing time, having rooms available in the clinic, having a table, etc., stand out in both. | In order to clarify some of the points contained within Table 2, the table was expanded and reorganized to minimize overlap. Due to the nature of subjective opinions, however, there will inherently remain some overlap in how these opinions are categorized. |
| **Conclusion** |  |
| 1. The data do not support the conclusion that patients aren’t aware. Patients were not interviewed in this study. In fact, one of the DOs interviewed stated that patients would accept OMT if it was explained to them what it does, in cases when the patient doesn’t know about it beforehand. So patient awareness may not be such a significant barrier. Certainly this study is not designed to assess patient awareness and thus it should not be part of the conclusion.  2. Patient awareness of OMT was not assessed in the interviews or surveys of the doctors and should not therefore be part of the conclusion which should be derived from the data gathered in this study. | The reviewer is absolutely correct. The intent was to express that these are challenges as reported by interviewees, but this was not clear in how it was phrased. The conclusion paragraph has been modified to reflect that these limitations are according to the opinions of the interviewees. |
| **General** |  |
| The remainder of the article is well conceived, written clearly, a pleasure to read, and the references are appropriate and up to date, other than the couple mentioned above. Methods were well articulated and referenced. The transcripts are the raw data and should be made available if another researcher wants to replicate or interpret the study in a different light, as the presentation of the data, the analyses and tables were anecdotal and thematic, with some examples selected by authors to make a point. Not sure if another researcher would come up with the same conclusions as these authors. Still, the article could benefit from some minor revisions as suggested above. It otherwise makes a scholarly addition to the literature on the subject. | The reviewer has made some excellent points that added to the clarity and rigor of the manuscript. We hope we have satisfactorily responded to the concerns. See comments below about making data available. |
| I am surprised that the study design was primarily a mixed method (see supporting information, IRB exempt : Full Study Title: “A Mixed Methods Analysis of Barriers to Osteopathic Manipulative Medicine”), but the authors only published the qualitative part. ICMJE recommends publishing all data and analysis of the same study in one paper, not in several ones. | The title of the IRB submission “Mixed Methods Analysis of Barriers to Osteopathic Manipulative Medicine” was chosen to match the title of the grant submission used to obtain funding. The funding supported several independent studies that shared a common research team and general topic of inquiry, but these studies were not unified to such a degree as to warrant presentation as a single manuscript. |
| **Editor Comments** |  |
| 1.Please ensure that your manuscript meets PLOS ONE's style requirements, including those for file naming. | We reviewed the style requirements and changed the format of the title page, headings, figures, and tables, including the figure file name. |
| 2. In this instance it seems there may be acceptable restrictions in place that prevent the public sharing of your minimal data. However, in line with our goal of ensuring long-term data availability to all interested researchers, PLOS’ Data Policy states that authors cannot be the sole named individuals responsible for ensuring data access. Data requests to a non-author institutional point of contact, such as a data access or ethics committee, helps guarantee long term stability and availability of data. Providing interested researchers with a durable point of contact ensures data will be accessible even if an author changes email addresses, institutions, or becomes unavailable to answer requests. Before we proceed with your manuscript, please also provide non-author contact information (phone/email/hyperlink) for a data access committee, ethics committee, or other institutional body to which data requests may be sent. If no institutional body is available to respond to requests for your minimal data, please consider if there any institutional representatives who did not collaborate in the study, and are not listed as authors on the manuscript, who would be able to hold the data and respond to external requests for data access? If so, please provide their contact information (i.e., email address). Please also provide details on how you will ensure persistent or long-term data storage and availability. | The following statement has replaced the former Data Availability statement: Participants did not give consent for broad distribution of their interviews. Researchers wishing to obtain access to the interview transcripts may contact the Mayo Clinic Health System regional research team at mchsswwires@mayo.edu. |
| 3. Your ethics statement should only appear in the Methods section of your manuscript. If your ethics statement is written in any section besides the Methods, please move it to the Methods section and delete it from any other section. Please ensure that your ethics statement is included in your manuscript, as the ethics statement entered into the online submission form will not be published alongside your manuscript. | The ethics statement was updated as follows: “This project was approved by the Mayo Clinic Institutional Review Board on June 16, 2023 (case ID, 23-004379). Recruitment of interviewees began on the same day. Funding was provided through a Mayo Clinic Health System Research Internal Award grant. Funding was used for research support staff, transcription services, and primary researcher protected time. Because interviews were conducted remotely via Zoom, interviewees provided verbal informed consent prior to participation. The use of verbal consent, including consent to use anonymized quotations in publications, was approved by the IRB. Interviewees were not compensated for their participation.” |
| 4. Please include captions for your Supporting Information files at the end of your manuscript, and update any in-text citations to match accordingly. | We did not submit any Supporting Information files. |
